# Ensemble of rankers for efficient gene signature extraction in smoke exposure classification

**DOI:** 10.1186/s12859-018-2035-3

**Published:** 2018-03-08

**Authors:** Maurizio Giordano, Kumar Parijat Tripathi, Mario Rosario Guarracino

**Affiliations:** High Performance Computing and Networking Institute (ICAR), National Council of Research (CNR), Naples, Italy

**Keywords:** Toxicology, Gene signature, Smoking, Supervised learning, Feature selection

## Abstract

**Background:**

System toxicology aims at understanding the mechanisms used by biological systems to respond to toxicants. Such understanding can be leveraged to assess the risk of chemicals, drugs, and consumer products in living organisms. In system toxicology, machine learning techniques and methodologies are applied to develop prediction models for classification of toxicant exposure of biological systems. Gene expression data (RNA/DNA microarray) are often used to develop such prediction models.

**Results:**

The outcome of the present work is an experimental methodology to develop prediction models, based on robust gene signatures, for the classification of cigarette smoke exposure and cessation in humans. It is a result of the participation in the recent sbv IMPROVER SysTox Computational Challenge. By merging different gene selection techniques, we obtain robust gene signatures and we investigate prediction capabilities of different off-the-shelf machine learning techniques, such as artificial neural networks, linear models and support vector machines. We also predict six novel genes in our signature, and firmly believe these genes have to be further investigated as biomarkers for tobacco smoking exposure.

**Conclusions:**

The proposed methodology provides gene signatures with top-ranked performances in the prediction of the investigated classification methods, as well as new discoveries in genetic signatures for bio-markers of the smoke exposure of humans.

**Electronic supplementary material:**

The online version of this article (10.1186/s12859-018-2035-3) contains supplementary material, which is available to authorized users.

## Background

System toxicology aims at understanding mechanisms, both at functional and genetic structural level, by which biological systems respond to toxicants. Such understanding can be leveraged to assess the risk of chemicals, drugs, and consumer products on living organisms. In particular, the identification of effective genomic biomarkers to aid prediction of toxicant/drug exposure levels in biological systems is an emerging research topic in system toxicology.

The increasing interest in this field is motivated by the wide applicability of genomic biomarkers for both finding evidence of toxicity in drug therapies and monitoring therapeutic outcomes. Furthermore, in case of acute poisoning, it can be used to detect exposure degree to toxicants/drugs. Indeed, the exposure level evaluation by safety biomarkers may lead to the development of more efficient diagnostic tools for toxicodynamic monitoring like in case of patients receiving immunosuppressive therapy [1]. This research area is relevant in many different applications, as shown by the identification of genomic biomarkers for a wide variety of toxicants, including nephrotoxic agents [2], testicular toxicants [3], for keratinocyte proliferation in papilloma murine skin model [4], and smoke exposure [5–7]. Several works propose the use of transcriptome-based exposure response signatures, computed by processing gene expression data (RNA/DNA microarray), to develop toxicant exposure prediction models [8–10]. In most of these approaches, gene signatures are identified by differential expression, using statistical tests involving case and control populations. Due to inter-individual variations present in human populations, observed gene sets could result in not-robust signatures. Indeed, robust signatures should maintain high specificity and sensitivity across independent subject cohorts, laboratories, and nucleic acid extraction methods.

In the present work we propose a methodology, as well as an experimental pipeline, for finding gene signatures for tobacco smoke exposure characterization and prediction. Our approach integrates different gene selection mechanisms, whose results are studied and compared to extract gene signatures more robust than those produced by a single methodology. In particular, the considered gene selection methods are based on a regression method (LASSO-LARS), a recursive elimination by support vector machines (RFE-SVM), and a feature selection by an ensemble of decision trees (Extra-Trees). While recent works start employing machine learning techniques for gene selection [11–13], the novelty of this work is to employ and merge the results from different gene selection methods, which are not limited to statistical analysis ones.

**Fig. 1:**
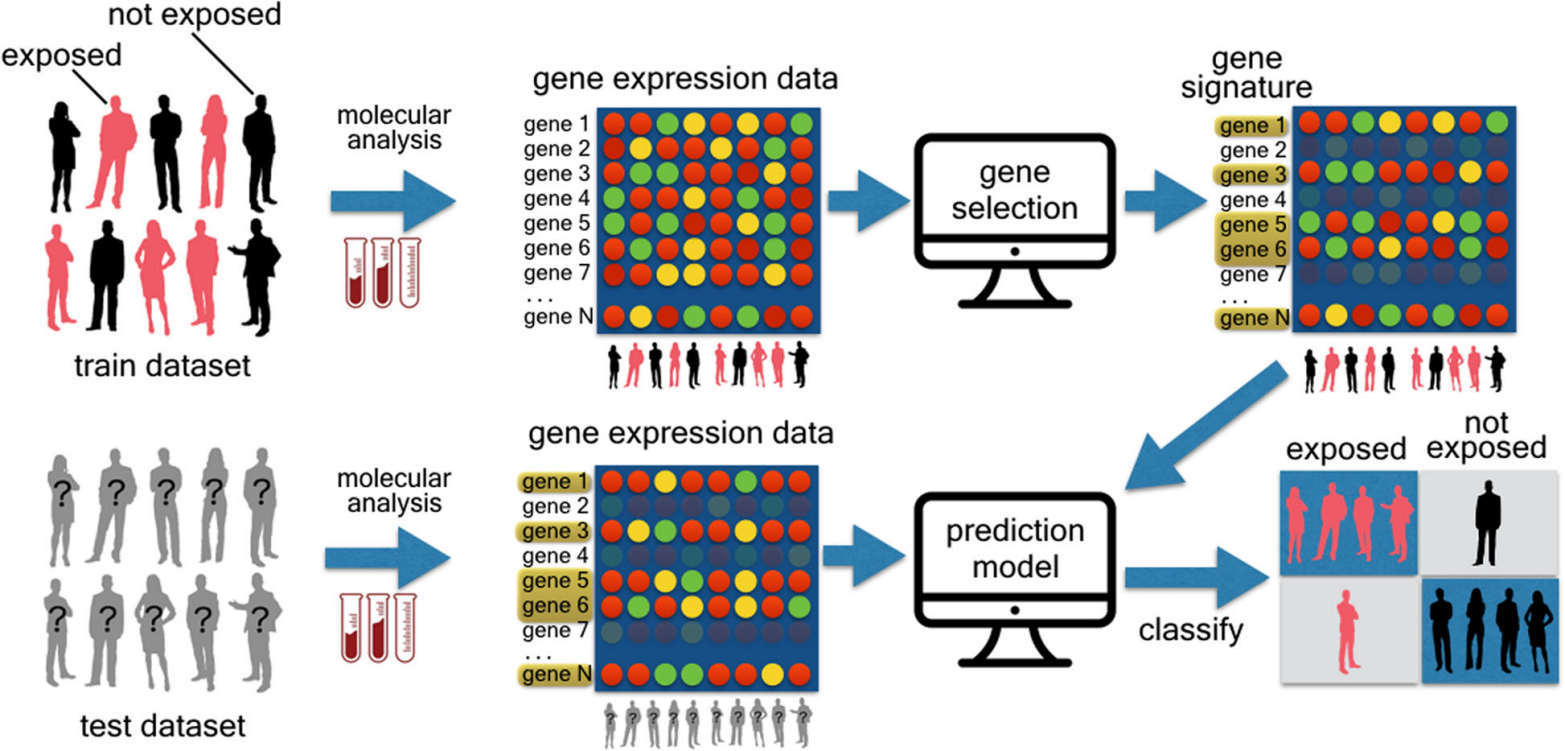
SysTox challenge workflow. First stage (up row): gene selection (signature) from the gene expression data from humans blood samples of the training dataset. Second stage (bottom row): develop inductive prediction models bases on training data from gene signature and provide classification results on testing dataset

The sbv IMPROVER project [14] is a collaborative effort led and funded by Philip Morris International Research and Development which focuses on the verification of methods and concepts in systems biology research within an industrial framework. sbv IMPROVER project has recently proposed the SysTox Computational Challenge [15] aiming at exploiting crowdsourcing as a pertinent approach to identify and verify chemical cigarette smoking exposure response markers from human whole blood gene expression data. The aim is to leverage these markers as a signature in computational models for predictive classification of new blood samples as part of the smoking exposed or non-exposed groups (see Fig. 1). In this application domain we investigated our methodology for gene expression data processing and selection as a machine learning problem of feature selection/reduction in a data space with high dimensionality (in the order of thousands of variables). In this context, we demonstrate how the blood gene signatures we found with our methodology have large overlaps with those found by other related works. In addition we identified new genes which are not mentioned in literature as possible biomarkers for tobacco smoke exposure. The functional annotation and terms enrichment analysis, together with toxicogenomics analysis (chemical-gene-disease-pathway association studies), showed that the expression levels of these new genes are affected by smoke exposure. In addition, based on our signatures we obtained higher performances in terms of area under precision-recall curve (AUPR) and matthews correlation coefficient (MCC) metrics by simply using a support vector machine (SVM) as a prediction model.

## Materials

In the SysTox Computation Challenge [15, 16] participants were asked to develop models to classify subjects as smokers versus non-current smokers (SvsNCS), and then former smokers versus never smokers (FSvsNS), based on the information from whole blood gene expression data from humans (subchallenge 1), or humans and rodents (subchallenge 2). The current investigation focuses only on tasks referring to subchallenge 1.

Figure 1 depicts the workflow of mandatory tasks the challengers were asked to follow. The workflow is the same as in the two classification problems proposed by the challenge. In the first stage of the challenge, a training dataset of gene expression data from human (or human/rodent) blood samples was made available for download to participants. The first task to be done was *gene selection* from whole blood gene expression data contained in the training dataset. The result of this task is a robust *gene signature* to be used to reduce training and testing data dimensions. Participants had also to develop *inductive prediction models* based on training data limited to the gene signatures they had previously identified. Inductive models are developed based only on training data. Classification on each test sample could be carried out only with the previously developed model, without retraining. Inductive models are different from *transductive* models in which training and testing datasets are processed together and used to retrain models prior to classification prediction. After all participants had submitted their results, in terms of both gene signatures and prediction models, the second stage of the challenge started: testing dataset of gene expression data from human (or human/rodent) blood samples were made available to participants. By using their proposed signatures and predictors, participants had to produce predictions (in terms of probabilities) on testing (unlabeled) samples.

After the competition closing, challenge organizers evaluated results submitted by participants only on a subset of testing samples which had been provided during the competition, the so called *gold labels*. Prediction models scores and rankings are reported on the sbv IMPROVER SysTox Challenge website.

Human blood sample data are organized in two datasets:

- *H1 training dataset*: a clinical case-control study conducted at the Queen Ann Street Medical Center (QASMC), London, UK and registered at ClinicalTrials.gov with the identifier NCT01780298 [5, 17]. The QASMC study aimed at identifying biomarkers to discriminate smokers with chronic obstructive pulmonary disease (COPD) (i.e., cigarette smoke with a ≥ 10 pack/year smoking history and COPD disease classified as GOLD Stage 1 or 2) from three groups of subjects which are matched by ethnicity, sex, and age (within 5 years) with the recruited COPD subjects: smokers (S), former smokers (FS), and never smokers (NS). All smoking subjects (S and FS) had a smoking history of at least 10 pack-years. FS quit smoking at least 1 year prior to sampling (∼ 78% of FS have stopped for more than 5 years). Patients included males (58%) and females (42%) aged between 40 and 70 years.
- *H2 testing dataset*: a transcriptomics dataset (BLD-SMK-01) produced from PAXgene ^*TM*^ blood samples obtained from a biobank repository (BioServe Biotechnologies Ltd., Beltsville, MD, USA) [5]. At the sampling time, the subjects were between 23 and 65 years of age. Subjects with a disease history and those taking prescription medications were excluded. Smokers (S) had smoked at least 10 cigarettes daily for at least three years. Former smokers (FS) quit smoking at least two years before the sampling and before cessation had smoked at least 10 cigarettes daily for at least three years. Smokers (S) and never smokers (NS) were matched by age and sex, while former smokers could not be properly matched due to the lower number of samples available for this group.

**Fig. 2:**
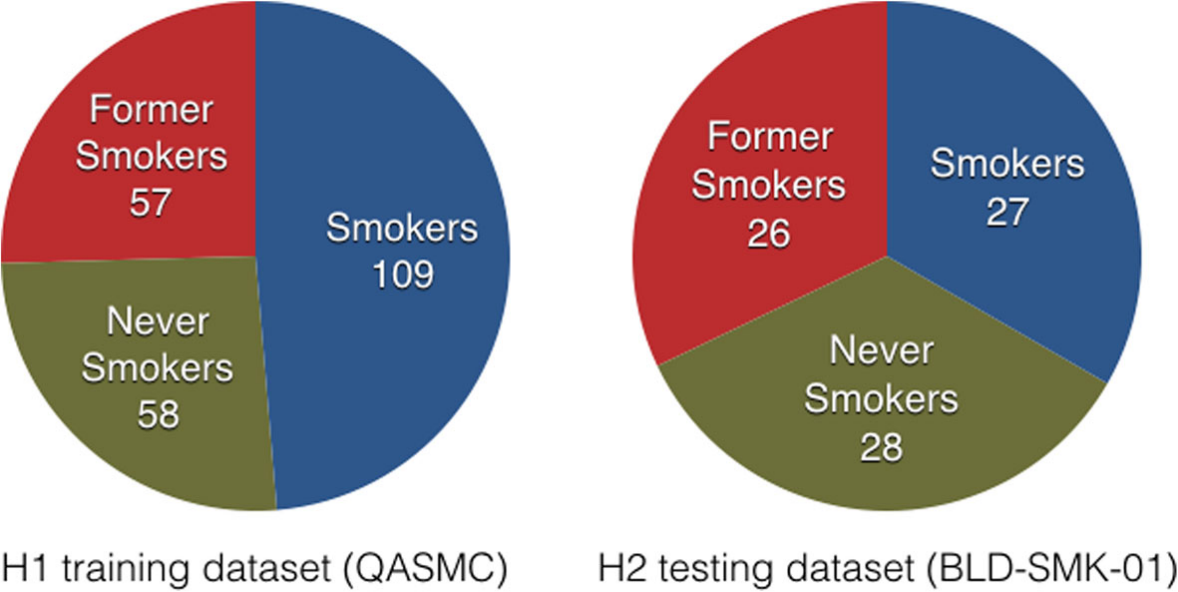
SysTox challenge datasets. Distributions of training labels and testing (gold) labels into classes of subjects: smokers (treated group), former smokers (cessation group), and never smokers (control group)

Sample data of H1 and H2 consist of DNA microarray experiments obtained with GeneChip Human Genome U133 Plus 2.0 Array and GeneChip Mouse Genome 430 2.0 Array (Affymetrix), on blood samples. Microarray data of both H1 and H2 are available in the ArrayExpress database [18], respectively under accession numbers E-MTAB-5278 and E-MTAB-5279. The distribution of training and testing labels and their categories are depicted in Fig. 2. For the human samples, 18604 gene expression data were provided.

## Methods

### Gene selection

The basic idea of our gene signature extraction approach is to identify an overlapping among the most discriminant genes we found out by applying three different feature selection techniques:

1. Feature selection by importances in forests of trees (Extra-Trees) [19]
2. Cross-validated Lasso, using the LARS algorithm [20]
3. Recursive Feature Elimination with SVM estimator [21]

Extra-Trees belong to the class of ensemble learning methods. They are based on bagging several instances of a black-box estimator (e.g. a decision tree) on random subsets of the original training set and then combining their individual predictions to form a final prediction. Bagging estimators is a very simple way to improve with respect to a single model without making it necessary to adapt the underlying base algorithm. In many cases, bagging methods reduce overfitting as well as the variance of a base estimator. In this work we use the feature selection facility of the Extra-Trees implementation available in the Python Scikit-learn [22].

LASSO (Least Absolute Shrinkage and Selection Operator) is a regression method performing feature selection by regularization of regression parameters (e.g. constraining the sum of their absolute values). The computation of the LASSO solutions is a quadratic programming problem, and can be tackled by standard numerical analysis algorithms that estimate sparse coefficients. It is widely recognized that the Least Angle Regression procedure (LARS) is the better approach since it exploits the special structure of the LASSO problem, and it provides an efficient way to compute the solutions simultaneously for all values of the regularization parameter. In this work we use the LASSO method with LARS algorithm for feature selection. In the remaining of the paper we will refer to this feature selection method as LASSO-LARS. In particular we use its implementation available in the Python Scikit-learn library.

Recursive Feature Elimination with SVM (RFE-SVM) By starting with the complete set of features, RFE-SVM repeats the following three steps until no more features are left: 1) train a SVM model; 2) compute a ranking of features as the squares of the hyperplane coefficients of the SVM model; and 3) remove the features with the worst ranks. In this work we use the RFE-SVM implementation available in Weka Data Mining Software [23].

The three methods produce as outputs three lists of ranked genes in reversal order. Regardless of the ranking criteria (respectively as Decision Treed importance scores, LASSO coefficient estimates, and SVM hyperplane coefficients) the three lists of genes are cut-off to the first hundred of genes with higher ranks.

### Prediction models

The focus of this work is on the data processing methodology to get a robust gene signature. The idea is that if the gene signature is biologically relevant, then classifiers will provide statistically significant results. Therefore, in order to assess the quality and robustness of our gene selection method, on the basis of signatures produced by it, we built a large set of prediction models exploiting well-known supervised learning techniques. We considered a set of nine classifiers, ranging from decision trees to support vector machines, from artificial neural networks to clustering and statistic methods. For the purpose, we used implementations of machine learning techniques available in the opensource Python Scikit-learn library [22]. The list of classifiers, their parameters setting and acronyms are reported in Table 1. All methods run in their default parameter configuration, since we were not interested in fine-tuning of each classifier.

**Table 1:** Prediction models

Classifier	Acronym	Parameters
Random forests	RF	split=gini, max depth=none, min samples leaf=1, min samples split=1, max features=auto, no. estimators=10
Gaussian Naive Bayes	GNB	*none*
*k*–Nearest neighbors	kNN	no.neighbors=3, algorithm=auto, metric=minkowski, p=2, weights=uniform, leaf size=30
MultiLayer perceptron	MLP	activation=relu;algorithm=l-bfgs, *α*=1e-05, *β*1=0.9, beta2=0.999, *ε*=1e-08, hidden layer sizes=(100,)
Support vector classifier	SVC	kernel=linear, C=0.1, tolerance=0.001
Logistic regression	LR	C=1.0 max iter=100 penalty=L2 tolerance=0.0001, multi class=OvR
Linear discriminant analysis	LDA	solver=SVD, tolerance=0.0001
Gradient tree boosting	GTB	loos=deviance, subsample=1.0 learning rate=0.1, min sample split=2, mean sample leaf=1, max depth=3, estimators=100
Extremely randomized trees	ERT	split=gini, max depth=No, min samples leaf=1, min samples split=1, max features=auto, no. estimators=10

The set of nine prediction models built by means of supervised learning on expression data (from H1 training dataset) of gene signatures

### Biological and toxicological interpretation of gene signatures

To understand the importance of gene signatures with respect to biological function and toxicological effects, we used Comparative Toxicogenomics Database (CTD) [24] and Transcriptator web-application [25] for the enrichment analysis of chemical association, diseases, pathways and gene ontology terms for our gene signatures. The CTD database is publicly available and provides knowledge about how environmental exposures affect human health. It contains both the curated and inferred information regarding chemical–gene/protein interactions, chemical–disease and gene–disease relationships. The functional gene ontology and pathway data related to genes are also included to study the possible mechanisms underlying environmentally influenced diseases. The curated information about gene-chemical interaction, gene-disease association and chemical-disease association is basically obtained through literature. Inferred relationships between gene-disease, gene-chemical and chemical-disease association are established via CTD. For example in case of gene-disease-chemical association network, gene A is associated with disease B because gene A has a curated interaction with chemical C, and chemical C has a curated association with disease B. The database provides inference scores for all inferred relationships. These scores reflect the degree of similarity between CTD chemical–gene–disease association networks and a similar scale-free random network. A high score, suggests a stronger connectivity. We obtained the chemical-gene-disease association information for all the gene signatures. Later we filter out genes only associated to “Tobacco smoke exposure” with inference score cutoff ≥ 50. We obtained the disease association, pathways enrichment and gene ontology enrichment for gene signatures and carried out comparison between them through set analysis using Venn diagram.

## Results and discussion

### Gene selection

Each feature selection technique has been applied to the datasets, in both SvsNCS and FSvsNS classification problems, by setting a limit to the maximum number of selected genes (one hundred). For each problem the three sets found have been intersected to find a robust gene signature.

**Fig. 3:**
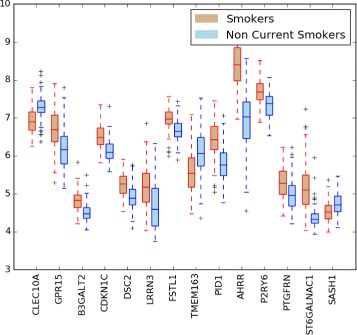
SvsNCS signature. Boxplot distribution of expression data (from H1 training dataset) of genes from the signature obtained for the case study of smokers versus non-current smokers

In the case of SvsNCS problem the results of the first hundred top-ranked genes by applying the three selection criteria are presented in Tables 2, 3 and 4. The three lists of genes show an overlap (the gene names in bold in the table) in the topmost positions. The set of 14 genes shared by all three lists form the resulting gene signature we propose for the SvsNCS case study. In Fig. 3 we have reported the boxplot of expression data in the training dataset of the 14-gene signature obtained with our approach.

**Table 2:** RFE-SVM SvsNCS signature

**AHRR**	**LRRN3**	**SASH1**	**CDKN1C**	SEMA6B	RAD52	**FSTL1**	**DSC2**	SYCE1L	**TMEM163**
CRACR2B	MOG	ZP4	KIT	**P2RY6**	AK8	PLA2G4C	MIR4697HG	SPAG6	ZNF618
**CLEC10A**	COL5A1	**B3GALT2**	TREM2	TYR	MMP3	LHX8	KCNJ2-AS1	**ST6GALNAC1**	SCIN
SPRY2	ADRA2A	GCNT3	PTGFR	PACRG-AS1	LINC00599	NR4A1	CHI3L1	TPPP3	SLC25A20
NT5C1A	TCEB3B	BMP7	FANK1	TMTC1	FGD5	APCDD1L	GYS2	TIMM8A	**PID1**
SHISA6	MYO1E	ADIRF-AS1	CTTNBP2	H19	P2RY12	DSTNP2	MAGI2-AS3	VSIG4	NR4A2
ICA1L	GFRA2	GSE1	NPIPB15	ZFP64	AFF3	FOXC2	CCR10	ARHGAP32	**GPR15**
RRNAD1	NOP9	HYPM	**PTGFRN**	SLC25A27	C3orf65	ZMYND12	TM4SF4	C6orf10	DUSP4
FUCA1	PALLD	ETNPPL	HMGCS2	LMOD3	EFNB1	FABP4	WNT2	FAM187B	LINC01270
PRKG2	NMNAT2	CYP4A11	FAM19A2	S1PR5	LINC00544	LRPAP1	CTSV	LOC200772	THBS2

Gene signature obtained with Recursive Feature ith SVM in in smokers versus non-current smoker case study. Gene names in bold are also present in the signatures found by Extra-Trees and LASSO-LARS methods

**Table 3:** Extra-Trees SvsNCS signature

**LRRN3**	LINC00599	**P2RY6**	**CDKN1C**	**GPR15**	**AHRR**	CTTNBP2	**DSC2**	**CLEC10A**	PF4
RGL1	**SASH1**	**FSTL1**	**PTGFRN**	C15orf54	MCOLN3	F2R	P2RY1	GUCY1A3	NRG1
SEMA6B	ESAM	CR1L	**PID1**	GP1BA	MAPK14	PBX1	GNAZ	GP6	**TMEM163**
RNASE1	SLC44A1	ASGR2	GUCY1B3	ZNF101	LTBP1	TRIP6	SRRD	PRR5L	CYSTM1
**B3GALT2**	GRAP2	ANKRD37	MKNK1	BEX2	SV2B	FAXDC2	**ST6GALNAC1**	ICOS	NFIB
TRDC	SLPI	CDK2AP1	IL4R	GPR20	SH2D1B	TLR5	VIL1	ITGB5	IGSF9B
CDR2	BTBD11	ELOVL7	ARL3	TUBB1	BZRAP1	ADAMDEC1	C2orf88	COCH	LOC100506870
LOC100130938	CA2	P2RY12	SH3BGRL2	PCSK6	PRTFDC1	SAMD14	CYP4A11	ASAP2	H19
LOC283194	BLCAP	GORASP1	TGM2	SLC26A8	ZAK	PARD3	MB21D2	GP9	S100A12
FANK1	TNFSF4	ZNF618	FAM210B	MYBPC3	SLC35G2	ASIC3	SLC6A4	CNST	PAPSS2

Gene signature obtained with feature selection of Extra-Trees in smokers versus non-current smoker case study. Gene names in bold are also present in the signatures found by RFE-SVM and LASSO-LARS methods

**Table 4:** LASSO-LARS SvsNCS signature

**CDKN1C**	**GPR15**	**LRRN3**	GPR63	**P2RY6**	**SASH1**	**CLEC10A**	**AHRR**	GSE1	ARHGAP32
**DSC2**	CRACR2B	PTGFR	LHX8	**FSTL1**	SYCE1L	APCDD1L	OTC	**PID1**	**PTGFRN**
**TMEM163**	CCR10	P2RY12	**B3GALT2**	**ST6GALNAC1**	RAD52	TRDC	BCLAF1	KNTC1	CLSTN3
ZNF536	ACAP1	DLGAP5	IFT140	LAPTM4A	MTSS1	SETD1A	CCP110	GPRASP1	USP34
SPCS2	PHACTR2	TM9SF4	HDAC9	SART3	BMS1	KIAA0232	DOCK4	TBC1D5	CEP104
PIEZO1	PTDSS1	VPRBP	SECISBP2L	SLK	FAM65B	KIAA0195	SNPH	EIF4A3	RAPGEF5
RASSF2	KIAA0101	JADE3	KIAA0247	ZFYVE16	KIAA0513	LZTS3	RIMS3	SNX17	MLEC
TOX	DHX38	RAB11FIP3	HDAC4	FRMPD4	KMT2B	TBKBP1	STARD8	ZSCAN12	RNF144A
ATG13	KIAA0586	PCDHA9	MATR3	NOS1AP	ZNF646	SDC3	KIAA0430	DZIP3	SAFB2
EIF5B	IPO13	WSCD2	SLC25A44	CEP135	KIAA0040	TTI1	PPIP5K1	PHF14	FAM53B

Gene signature obtained with Least Absolute Shrinkage and Selection Operator (with Least Angle Regression procedure) in smokers versus non-current smokers case study. Gene names in bold are also present in the signatures found by RFE-SVM and Extra-Trees methods

**Fig. 4:**
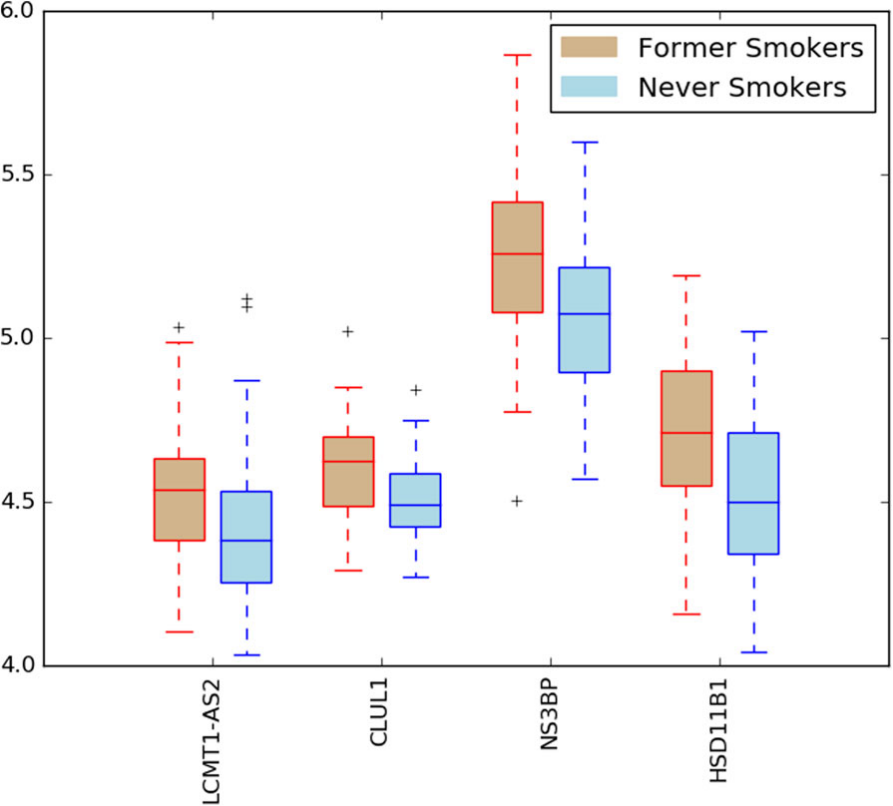
FSvsNS signature. Boxplot distribution of expression data (from H1 training dataset) of genes from the signature obtained for the case study of former smokers versus never smokers

In the case of FSvsNS problem, the results of the first hundred top-ranked genes by applying the three selection criteria are presented in Tables 5, 6 and 7. In this case a small overlapping exists between the three lists of genes produced by the three selection criteria. In particular, only 4 genes are shared (the gene names in bold in the table). The set of 4 genes shared by all three lists form the resulting gene signature we propose for the FSvsNS case study. In Fig. 4 we have reported the boxplot of expression data in the training dataset of the 4-gene signature produced by our approach. The experiments showed that by removing the gene LCMT1-AS2 we obtained a more robust gene signature.

**Table 5:** RFE-SVM FSvsNS signature

SLC38A3	POU4F1	**HSD11B1**	GOLGA2P5	IL17RD	CELF5	ADAMTS14	PTPN14	MB21D2	TBC1D29
RRP12	C4BPB	KRT73	DCAF4	ZNF280B	LOC648691	DDX11	TJP3	LINC01097	BCL2L12
RAB42	CLSPN	ADAM23	CFD	TAS2R9	CFAP46	VSIG4	GDF9	SI	DOCK4-AS1
SH3PXD2A-AS1	**CLUL1**	MMP1	PLA2G2A	RTN3	LY6G6D	ANKRD6	IGSF9B	ZNF582-AS1	C8orf88
REG3A	ETV2	NDST3	C6orf99	WNT5B	PAX4	NNAT	HCG26	SLC5A11	TAAR3
TTC22	HAGHL	C17orf78	EDN2	MTUS1	PLCD4	C1orf115	PLEK	**NS3BP**	SLC34A2
GGT5	ZNF470	SYN1	SCD	MRAS	FOXI1	**LCMT1-AS2**	HTN3	SH3D19	HIST1H4E
SHISA6	MCOLN3	LOC100507534	SASH1	APEX1	C22orf31	RNF114	SRRM4	SCN2B	HMBOX1
ATP6V1C2	HSF4	SLC17A5	SEPT2	TFAP4	WWTR1	FGF4	SRCIN1	SLC35F1	SLC16A2
TAS2R50	PCAT19	ADAMTS18	TMEM31	CAMK1G	SLC25A31	SMR3B	SLC17A4	XRCC6BP1	PTPRB

Gene signature obtained with Recursive Feature Elimination with SVM in former smokers versus never smokers case study. Gene names in bold are also present in the signatures found by Extra-Trees and LASSO-LARS methods

**Table 6:** Extra-Trees FSvsNS signature

MMP1	PRR29	APCS	**HSD11B1**	DLK2	**NS3BP**	CNTN2	CLDN17	CHGA	TMEM31
MAPK10	ZNF280B	C20orf85	LDHD	**CLUL1**	MAF	WFIKKN2	CYP4B1	NTRK3-AS1	NKX6-1
FAM221A	IFIT1	SLC16A1	HSD11B1L	**LCMT1-AS2**	CLCN1	IGSF9B	CENPU	ZNF652	GPAM
ENTPD7	FBXL19-AS1	PRKCE	HCG26	NLRP14	B3GNT7	KLF14	SLCO4A1	SNCG	SLC34A2
CEP76	CXorf36	ATF2	STAU2-AS1	SIGLEC11	RWDD3	ASB16	FGB	HIST1H4H	ERN2
CLRN1-AS1	SLC50A1	DOK4	FASTKD1	MB21D2	HDAC1	KIF2A	GMIP	CT83	CYP2A13
MED6	CHDC2	FGF13-AS1	IFNA21	DEPDC5	CEP250	MCM3AP	KRT75	GLP1R	RAD51B
CFAP20	TMEM184A	HOMEZ	LINC00922	CRP	MAST1	CBL	SDF4	KRT19	CELF5
CDCA8	ACTL8	MRPS12	ACER1	SYCE3	AP4E1	TYK2	LOC283914	SLC12A1	SCN2A
PLAC4	OXCT1	ABCA11P	GLB1	TCEAL7	LRRC32	BHLHE22	LINC01012	TBK1	TMEM225

Gene signature obtained with feature selection of Extra-Trees in former smokers versus never smokers case study. Gene names in bold are also present in the signatures found by RFE-SVM and LASSO-LARS methods

**Table 7:** LASSO-LARS FSvsNS signature

POU4F1	PTPRB	**CLUL1**	SLC38A3	PTPN14	GDF9	**LCMT1-AS2**	C4BPB	LINC00901	**HSD11B1**
HSF4	ADAMTS18	SEPT2	LOC648691	EDN2	LINC00319	DOCK4-AS1	TMEM246	PBK	LINC00964
SLC7A11	IL17RD	TBC1D29	PTPN3	**NS3BP**	KIAA0513	KIAA0586	IFT140	LAPTM4A	RNF144A
MATR3	RIMS3	SETD1A	CCP110	GPRASP1	USP34	SNX17	DHX38	KNTC1	HDAC9
PIEZO1	SART3	DOCK4	CEP104	VPRBP	SECISBP2L	RAB11FIP3	ZNF646	TMEM63A	UTP14C
SEMA3E	NOS1AP	GPRIN2	ARHGAP32	ACAP1	ZFYVE16	PCDHA9	KIAA0247	LZTS3	MLEC
TOX	HDAC4	FRMPD4	JADE3	KMT2B	TBKBP1	KIAA0101	STARD8	ZSCAN12	SNPH
ZNF536	FAM65B	RASSF2	RAPGEF5	SLK	KIAA0195	BCLAF1	EIF4A3	ATG13	TM9SF4
CLSTN3	KIAA0232	TBC1D5	PHACTR2	KIAA0226	ADAMTSL2	KIAA0430	MDC1	IQCB1	ZNF516
PDE4DIP	CEP135	LPIN2	DZIP3	TTLL4	SAFB2	EIF5B	IPO13	WSCD2	SDC3

Gene signature obtained with Least Absolute Shrinkage and Selection Operator (with Least Angle Regression procedure) in former smokers versus never smokers case study. Gene names in bold are also present in the signatures found by RFE-SVM and Extra-Trees methods

### Signatures biological interpretation

With respects to the SvsNCS problem, the lists of the first hundred of top-ranked genes are reported in Tables 2, 3 and 4. As we may note, these gene lists share 14 genes which are associated to very high ranks in all of them.

**Fig. 5:**
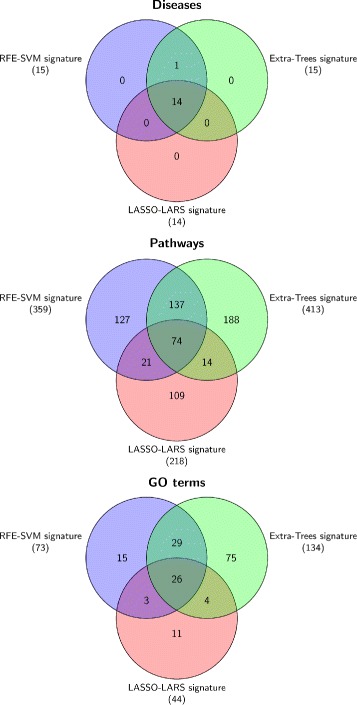
Diseases-pathways-GO-terms association to SVM, Extra-Trees and LASSO-LARS signature. Comparative analysis of gene-disease-pathways-gene ontology terms associated to the gene signatures which were obtained with RFE-SVM, Extra-Trees and LASSO-LARS selection methods in the case study of smokers versus non-current smokers

To analyze these signatures, we obtained the gene-chemical association results from CTD database and we selected genes which interact with tobacco smoke pollution with higher inference score. Later, we carried out inferred gene-disease association, pathways and gene ontology enrichments analysis. The results are provided in the supplementary tables reported, in the ‘Additional files’ section, from ‘Additional files 1, 2 and 3’. The comparative analysis of disease association, pathway and gene ontology terms enrichment of the signatures obtained with the three gene selection techniques (Extra-Trees, LASSO-LARS and RFE-SVM), provide a clear and robust picture of the signature associated with smoking effects. From our analysis (Fig. 5), we infer that though the overall overlap between the gene signatures from these methods is small, yet the gene signatures from the three methods shares a good amount of gene-disease association and most of these genes are involved in the same diseases.

We also observed that the diseases associated to these genes are respiratory tract, pregnancy complications, cardio-vascular, neoplasm, fetal disorder, congenital abnormalities, endocrine system diseases. Similarly, these genes share 74 common pathways, and some of these pathways (cell cycle, chemokine receptors bind chemokines, cytokine signaling in immune system, cytokine-cytokine receptor interaction, mitotic G1-G1/S phases, platelet activation, signaling and aggregation, post-translational protein modification, PPARA activates gene expression, Rap1 signaling pathway and Ras signaling pathway) are known to be involved in cancer progression.

The gene ontology enrichment and comparative analysis also suggest that most of these genes are involved in protein binding, membrane, localization, ion binding, regulation of biological process and signal transduction. In the light of these results, we deduce that the three gene signatures produced by our selection criteria, with respect to the smokers versus non-current smokers case study, although different still share the same biological and toxicological characteristics. The overlap analysis among the three methods reported more stronger gene signature. We selected the genes common to all three methods and carried out the enrichment analysis.

The enrichment analysis of the gene signature we identified for the SvsNCS problem shows that all 14 genes are enriched (see Table 8) in biological processes, such as cellular response to chemical stimulus, and in molecular functions, such as protein binding, ion binding, molecular transducer activity.

**Table 8:** SvsNCS signature biological interpretation

Gene name	Gene description	Chemical interaction
CLEC10A	C-type lectin domain containing 10A	Benzo(a)pyrene
GPR15	G protein-coupled receptor 15	Tobacco Smoke Pollution
B3GALT2	beta-1,3-galactosyltransferase 2	Tobacco Smoke Pollution, Tretinoin, Valproic Acid, Vehicle Emissions
CDKN1C	cyclin-dependent kinase inhibitor 1C (p57, Kip2)	Tetrachlorodibenzodioxin, tert-Butylhydroperoxide, Valproic Acid
DSC2	desmocollin 2	Tetrachlorodibenzodioxin, Valproic Acid
LRRN3	leucine rich repeat neuronal 3	Tobacco Smoke Pollution
AHRR	aryl-hydrocarbon receptor repressor; programmed cell death 6	Benzo(a)pyrene
TMEM163	transmembrane protein 163	Valproic Acid, Benzo(a)pyrene
PID1	phosphotyrosine interaction domain containing 1	Valproic Acid, Benzo(a)pyrene
FSTL1	follistatin-like 1	Methylnitronitrosoguanidine co-treated with Cadmium Chloride
P2RY6	pyrimidinergic receptor P2Y, G-protein coupled, 6	Benzo(a)pyrene
PTGFRN	prostaglandin F2 receptor inhibitor	Benzo(a)pyrene, Tetrachlorodibenzodioxin, Valproic Acid
ST6GALNAC1	ST6 N-acetylgalactosaminide alpha-2,6-sialyltransferase 1	Acetaminophen, Clofibrate, Phenylmercuric Acetate
SASH1	SAM and SH3 domain containing 1	Benzo(a)pyrene

Enrichment analysis of the proposed gene signature in the smokers versus non-current smokers case study

It is worth to notice that 4 genes from the proposed gene signature were already known in literature as biomarkers for cigarette smoke exposure. Indeed, genes LRRN3, SASH1, TNFRSF17, CDKN1C have been studied in [5], while LRRN3 gene was already known as biomarker in [26]. These genes were also found as biomarkers by the three winning teams participating in the SysTox Computational Challenge. Moreover these genes occupy the first positions in all the signatures that we identified. This is a further confirmation that our gene ranking criteria are in agreement with other approaches published in literature.

Similarly, we obtained the gene signatures for FSvsNS case study, by applying RFE-SVM, Extra-Trees and LASSO-LARS selection methods. The gene signatures are provided in Tables 5, 6 and 7 and they share only four genes.

In case of former smoker versus never smokers study, the enrichment analysis of the found gene signature shows that three genes which are included in our signatures (ADAMTS14, SLC38A3, HSD11B1), are known to contain SNPs or somatic mutations and differential expressed in lung/bladder cancers. The toxicogenomics gene-chemical-disease association study and the resulting biological and toxicogenomics data are provided in the supplementary tables reported, in the ‘Additional file’ section, from ‘Additional files 4, 5, 6’.

Table 9 shows the overlapping matrix of the gene signature resulting from our method with genes signatures produced by Philip Morris International (PMI) and by the three winning teams of the challenge (T264, T225 and T259) [27]. As we can see, in the overlap matrix our signature shares 8 out of 14 genes with the three teams (CLEC10A, GPR15, CDKN1C, LRRN3, AHRR, PID1, P2RY6, and SASH1). The remaining 6 genes (B3GALT2, DSC2, TMEM163, FSTL1, PTGFRN and ST6GALNAC1) were neither found by PMI nor by the winning teams. In the remaining of the document we will refer to the set of 8 genes shared by the three winning teams of the challenge as the *common gene signature*, while the set of 6 genes proposed only by us will be referred as *specific gene signature*. The completed set of 14 genes resulting from our method is referred as *total gene signature*.

**Table 9:** Signature overlaps among methods

Gene	Our	PMI	T264	T225	T259
CLEC10A	✓		✓	✓	✓
GPR15	✓		✓	✓	✓
B3GALT2	✓				
CDKN1C	✓	✓	✓	✓	✓
DSC2	✓				
LRRN3	✓	✓	✓	✓	✓
AHRR	✓		✓	✓	✓
TMEM163	✓				
PID1	✓		✓	✓	✓
FSTL1	✓				
P2RY6	✓		✓	✓	✓
PTGFRN	✓				
ST6GALNAC1	✓				
SASH1	✓	✓	✓	✓	✓
RGL1		✓		✓	✓
SEMA6B			✓	✓	✓
CTTNBP2			✓	✓	
F2R			✓	✓	

Overlap matrix of the proposed gene signature with those produced by PMI and by the three winning teams of the SysTox Computational Challenge (for the smokers versus non-current smokers case study)

**Fig. 6:**
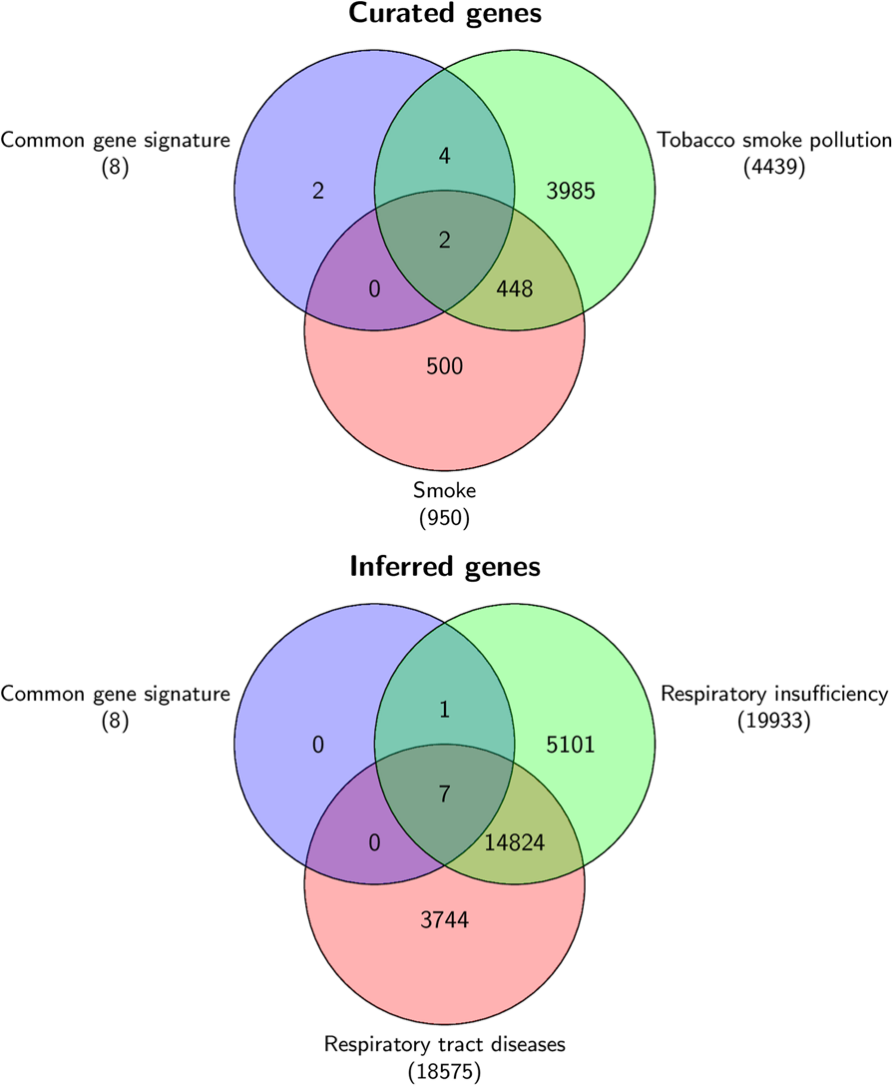
Disease-chemical association of common gene signature. Disease and chemical association of 8 genes (common gene signature) from our signature which are shared by the three winning teams of the challenge (smokers versus non-current smokers case study)

**Fig. 7:**
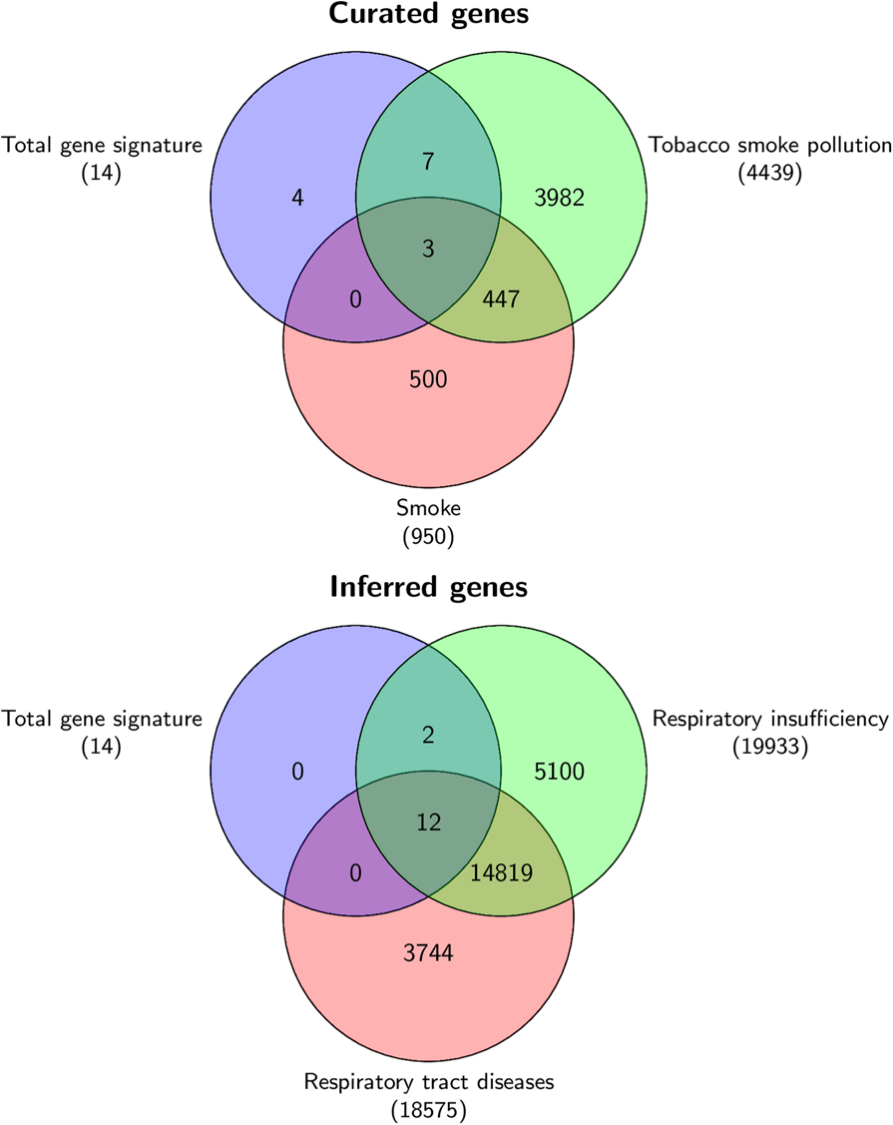
Disease-chemical association of total gene signature. Disease and chemical association of our signature which includes 6 genes not-shared by the three winning teams of the challenge (smokers versus non-current smokers case study)

We focused on these genes and carried out gene-chemical-pathways association studies using CTD database. The results are showed in Figs. 6 and 7 and in the supplementary tables reported, in the ‘Additional files’ section, from ‘Additional files 7, 8, 9, 10, and 11’. Interestingly, we observe in Fig. 6 that the common gene signature has stronger affinity for smoke, tobacco smoke and Benzo(a)pyrene, the later being a constituent of cigarette smoke. By including in the analysis the 6 genes found only by us, we observe in Fig. 7 that the total gene signature still shows a stronger affinity for smoke and tobacco smoke.

We also determined the disease association for these 14 genes with inference score greater than threshold (≥ 50) with respect to respiratory tract disease and respiratory insufficiency. Both these diseases of respiratory tract are well characterized in literature as a negative result of tobacco smoking.

We also carried out the pathways enrichment analysis for both the common gene signature and the specific gene signature in the case study of smokers versus non-current smokers. Biological and toxicogenomics analysis suggest that these 6 genes specific to our analysis are also very interesting with respect to smoking and could be further investigated as potential biomarkers for tobacco smoking exposure.

**Fig. 8:**
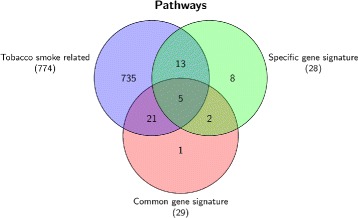
Pathways overlap in pathways dataset. Overlap of pathways information of common (8) and specific (6) gene signatures (obtained for the case study of smokers versus non-current smokers) with tobacco smoking exposure related complete pathways dataset

On comparing the enriched pathways in both common and specific gene signature with respect to the whole set of pathways associated with tobacco smoking, we determined the significant overlapped pathways for these 14 genes. Some of the main pathways are Class A/1 (rhodopsin-like receptors), GPCR downstream signaling, GPCR ligand binding, signal transduction and signaling by GPCR. The results are shown in Fig. 8. Out of 28 enriched pathways in specific gene signatures and 29 pathways in common gene signature, 18 and 26 pathways in both the signatures sets are effected by tobacco smoke. Most of these tobacco smoking associated pathways are involved in biological pathways such as cell signaling, platelet activation signaling and aggregation, post-translational protein modification, signaling by BMP, developmental biology, cell cycle, mitotic cyclin D associated events in G1, fatty acid, triacylglycerol and ketone body metabolism, G alpha (q) signalling events, innate immune system, metabolism, metabolism of lipids and lipoproteins and mitotic G1-G1/S phases. All these pathways are associated with the proper functioning of the cell.

The tabular results of pathways information associated with common and specific gene signature as well as the overlap analysis with tobacco smoking is provided in the ‘Additional file 11’. Biological interpretation of these gene signatures using information from CTD database helps in the strengthening of our prediction model. More interestingly, we obtained a greater number of genes in our signature for smoker versus non-current smokers case study. The 6 genes which are not reported by other participants of the challenge, but suggested by our method, are also interesting and share the same biological and toxicological properties as the other genes of the signatures shared by the other participants. By taking into account these additional genes in our prediction model, we do have better chance to characterize smokers versus non-current smokers and surely this help in strengthening our prediction models over those proposed by the challengers.

With regards to the former smoker versus never smokers classification problem, we compared the gene signatures from the three selection methods and extracted three overlapping genes: CLUL1, NS3BP and HSD11B1. Biological and toxicological analysis of these three genes (see Table 10) suggests their chemical associations with valproic acid and tetrachlorodibenzodioxin. The later chemical is usually formed as a side product in organic synthesis and burning of organic materials and is a carcinogenic in nature. CLUL1 is involved in “Prenatal Exposure Delayed Effects” due to its chemical interactions with tetrachlorodibenzodioxin and bisphenol A. HSD11B1 is also involved in “Prenatal Exposure Delayed Effects” and it is also known to have chemical interactions with tetrachlorodibenzodioxin and bisphenol A.

**Table 10:** FSvsNS signature biological interpretation

Gene name	Gene description	Chemical interaction
CLUL1	clusterin like 1	Valproic Acid, bisphenol A
NS3BP	NS3 binding protein	*Not Available*
HSD11B1	hydroxysteroid 11-beta dehydrogenase 1	Hydrocortisone, bisphenol A, Tetrachlorodibenzodioxin

Enrichment analysis of the proposed gene signature in the former smokers versus never smokers case study

### Prediction models

Once the datasets for both SvsNCS and FSvsNS classification problems were reduced in such a way to contain only expression data of genes beloning to our signatures, we started a set of experiments with different classification methods. For the experiments we chose a subset of classifiers available in the Python Scikit-learn package. The list of classifiers, their parameters settings and acronyms are reported in Table 1.

For both classification problems, we trained the classifiers on the H1 training dataset shrunk to the signature data. This supervised training procedure yielded to the construction of inductive prediction models for the two case studies. Later, the built models were used to classify (gold) samples from the H2 testing dataset, which of course had been previously reduced to the signature data.

With respect to the smokers versus non-current smokers classification problem, the prediction results of the nine selected classifier, in terms of AUPR and MCC scores, are summarized in Table 11. The table reports also the scores obtained by the three winners of the challenge (T264, T225 and T259) for comparison. As we can see, the SVC classifier provided the best prediction performance (in both AUPR and MCC metric).

**Table 11:** Performance of classifiers using SvsNCS signature

	RF	GNB	kNN	MLP	SVC	LR	LDA	GTB	ERT	T264	T225	T259
AUPR	0.961	0.938	0.9140	0.9043	**0.9746**	0.9537	0.9484	0.9650	0.9580	0.96	0.97	0.95
MCC	0.9012	0.8766	0.8025	0.8272	**0.9259**	0.8148	0.8765	0.9136	0.8642	0.90	0.77	0.79

Performance measures, in terms of AUPR and MCC scores, of nine classifiers using the signature obtained for the case study of smokers versus non-current smokers. Results are compared to the scores obtained by winners of SysTox Computational Challenge. Best results in boldface

With respect to the former smokers versus never smokers classification problem, the AUPR and MCC scores of the selected classifiers are summarized in Table 12. As before, the table compares our results to the scores obtained by the three winners of the challenge. In this second case study, our results are more impressive, since the prediction scores are far better than those obtained by the other challengers.

**Table 12:** Performance of classifiers using FSvsNS signature

	RF	GNB	kNN	MLP	SVC	LR	LDA	GTB	ERT	T264	T225	T259
AUPR	0.6366	0.6357	0.6594	0.6710	**0.7321**	0.7024	0.6581	0.5528	0.6774	0.58	0.50	0.47
MCC	0.0845	0.1092	0.1310	0.0307	**0.2883**	0.2318	0.1472	-0.0644	0.1092	0.07	0.02	-0.02

Performance measures, in terms of AUPR and MCC scores, of nine classifiers using the signature obtained for the case study of former smokers versus never smokers. Results are compared to the scores obtained by winners of SysTox Computational Challenge. Best results in boldface

## Conclusions

The focus of this work is our contribution to the crowdsourcing initiative, namely the SysTox Computational Challenge, proposed by sbv IMPROVER project. The challenge initiative aims at identifying by crowdsourcing chemical cigarette smoking exposure biomarkers from human whole blood gene expression data.

In this context, this work proposed a methodology, as well as an experimental pipeline, to extract robust gene signatures from whole blood gene expression data. In addition, this work showed how to build predictive models based on robust gene signatures. Our models discriminate smokers from non-current smokers, as well as former smokers from never smokers subjects. In our computational approach we crossed three very different gene selection techniques to obtain robust gene signatures. Later, in order to assess the quality and robustness of the found gene signatures, we build, on the basis of expression data of selected genes of our signatures, nine prediction models implemented with different supervised machine learning techniques.

With regards to the SvsNCS classification problem we obtained high scores for the majority of the explored learning techniques, with AUPR and MCC scores comparable to (even better than) those obtained by the SysTox Challenge winners. Surprisingly, for what concerns the FSvsNS classification problem, the prediction models build on the basis of the found signatures performed far better than those proposed by the challenge winners.

The results obtained by our computational approach are strengthened by the functional annotation terms enrichment analysis, as well as by the toxicogenomics analysis (chemical-gene-disease-pathway association studies) for both the SvsNCS and FSvsNS gene signature. In case of SvsNCS, we obtained highly enriched functional terms such as regulation of steroid genesis, orphan nuclear receptors, nerve growth factor, DNA damage, signal transduction, and membrane associated terms. In the present understanding of negative effects of cigarette smoking on humans, the enriched terms and related genes are known to be associated with either cancer progression or nervous system. On the other hand, in case of FSvsNS, the enriched biological terms are generally associated with inflammatory response, extracellular regions, disulfide bonding. As expected, there are not such harmful effects observed in former smoker when compared to never smokers. The interesting observation about this list is that some of these genes such as ADAMS14, SLC38A3, HSD11B1 accommodate structure variation (SNPs) due to tobacco smoking exposure for longer period of time frame.

## Additional files

Additional file 1 Gene-disease-chemical study of Extra-Trees signature in SvsNCS. Gene-disease-chemical association studies for gene signature predicted by Extra-Trees method for smokers versus non-current smokers case study. (CSV 59 kb)

Additional file 2 Gene-disease-chemical study of LASSO-LARS signature in SvsNCS. Gene-disease-chemical association studies for gene signature predicted by LASSO-LARS method for smokers versus non-current smokers case study. (CSV 30 kb)

Additional file 3 Gene-disease-chemical study of RFE-SVM signature in SvsNCS. Gene-disease-chemical association studies for gene signature predicted by RFE-SVM method for smokers versus non-current smokers case study. (CSV 50 kb)

Additional file 4 Gene-disease-chemical of Extra-Trees signature in FSvsNS. Gene-disease-chemical association studies for gene signature predicted by Extra-Trees method for former smokers versus never smokers case study. (CSV 44 kb)

Additional file 5 Gene-disease-chemical of LASSO-LARS signature in FSvsNS. Gene-disease-chemical association studies for gene signature predicted by LASSO-LARS method for former smokers versus never smokers case study. (CSV 15 kb)

Additional file 6 Gene-disease-chemical of RFE-SVM signature in FSvsNS. Gene-disease-chemical association studies for gene signature predicted by RFE-SVM method for former smokers versus never smokers case study. (CSV 32 kb)

Additional file 7 GO-enrichment of common gene signature. Gene ontology enrichment analysis for 8 genes in common with other participants of the SysTox Computational Challenge. (CSV 11 kb)

Additional file 8 GO-enrichment of specific gene signature. Gene ontology enrichment analysis for 6 genes not in common with other participants of the SysTox Computational Challenge. (CSV 9 kb)

Additional file 9 Pathways-enrichment of common gene signature. Pathways enrichment analysis for 8 genes in common with other participants of the SysTox Computational Challenge. (CSV 2 kb)

Additional file 10 Pathways-enrichment of specific gene signature. Pathways enrichment analysis for 6 genes not in common with other participants of the sbv IMPROVER SysTox Computational Challenge. (CSV 2 kb)

Additional file 11 Pathways mapping of common versus specific gene signature. Mapping pathways enrichment for both common and specific gene signature with respect to the complete pathways set associated with tobacco smoke pollution. (CSV 93 kb)

## Abbreviations

AUPR: Area under precision-recall curve
ERT: Extremely randomized trees
FS: Former smokers
FSvsNS: Former smokers versus never smokers
GNB: Gaussian Naive Bayes
GTB: Gradient tree boosting
kNN: k–nearest neighbors
KPT: Kumar Parijat Tripathi (author)
LARS: Least angle regression
LASSO: Least absolute shrinkage and selection operator
LASSO-LARS: LASSO with LARS
LDA: Linear discriminant analysis
LR: Logistic regression
MCC: Matthews correlation coefficients
MG: Maurizio Giordano (author)
MLP: Multilayer perceptron
MRG: Mario Rosario Guarracino (author)
NS: Never smokers
PMI: Philip morris international
QASMC: Queen ann street medical center
RF: Random forests
RFE-SVM: Recursive feature elimination with support vector machines
S: Smokers
sbv IMPROVER: Systems biology verification combined with industrial methodology for process verification in research
SVC: Support vector classifier
SVM: Support vector machine
SvsNCS: Smokers versus non-current smokers
SysTox: Systems toxicology
